# Induction of M1 polarization in BV2 cells by propofol intervention promotes perioperative neurocognitive disorders through the NGF/CREB signaling pathway: an experimental research

**DOI:** 10.1097/JS9.0000000000002257

**Published:** 2025-01-28

**Authors:** Ting Ye, Yiwei Fan, Xiangye Zeng, Xiaojing Wang, Huaping Xiao

**Affiliations:** a Department of Anesthesiology, Jiangxi Cancer Hospital & Institute, Jiangxi Clinical Research Center for Cancer, The Second Affiliated Hospital of Nanchang Medical College, Jiangxi Key Laboratory of Oncology, Nanchang, Jiangxi Province, China; b Department of Anesthesiology, the First Affiliated Hospital, College of Medicine, Zhejiang University, Hangzhou, Zhejiang Province, China; c Nanchang University Jiangxi Medical College, Nanchang, Jiangxi Province, China

**Keywords:** CREB, microglial cell, neuroinflammation, NGF, PND, propofol

## Abstract

Nerve growth factor (NGF) is critical in regulating the homeostasis of microglial cells. It activates various signaling pathways that mediate the phosphorylation of cAMP response element-binding protein (CREB) at key regulatory sites. The decrease in phosphorylated CREB (p-CREB) expression is linked to neuroinflammatory responses. The exact molecular mechanism by which propofol regulates microglial polarization and induces neuroinflammation via the NGF/CREB signaling axis remains unclear. This study aims to investigate the specific mechanisms by which propofol induces perioperative neurocognitive disorders through microglial M1 polarization and neuroinflammation via the NGF/CREB signaling pathway. We demonstrated that propofol impairs neurocognitive function in mice, as evidenced by behavioral deficits. It reduces NGF expression in hippocampal microglia and BV2 cells, where protein-protein interactions between NGF and CREB suggest that NGF primarily regulates neurocognitive function by modulating p-CREB. Propofol intervention and inhibition of the NGF/CREB pathway promote M1 polarization in hippocampal microglia and BV2 cells, leading to reduced cell proliferation, increased apoptosis, elevated oxidative stress, and higher levels of the inflammatory marker TNF-α. Exogenous NGF does not alter the expression of NGF or total CREB but significantly upregulates p-CREB, indicating its regulatory role in signaling pathways associated with microglial activation. Moreover, exogenous NGF mitigates propofol-induced cognitive impairments and M1 polarization, reducing apoptosis and oxidative stress levels. Our findings suggest that propofol downregulates the expression of NGF and CREB, subsequently reducing p-CREB levels. This downregulation induces M1 polarization of microglia, promoting the progression of neuroinflammation and contributing to the development of perioperative neurocognitive disorders.

## Introduction

Perioperative neurocognitive disorders (PND) are common complications in elderly patients after surgery, with neuroinflammation playing a central role in the onset and progression^[1]^. Microglia, making up 10%–15% of brain cells, serve a critical immune defense role in the central nervous system^[2]^. Under normal circumstances, these cells are inactive, monitoring immune status and releasing neurotrophic factors such as nerve growth factor (NGF) and brain-derived neurotrophic factor to support neuronal growth, maintenance, and survival^[3]^. However, microglia are activated when surgical procedures, trauma, or infections stimulate the central nervous system, producing neurotoxic M1 phenotypes and neuroprotective M2 phenotypes^[4,5]^. An imbalance in the M1/M2 ratio due to overactivation of M1 microglia exacerbates neuroinflammation and impairs cognitive functions^[6]^. The activation of microglia and neuroinflammation contributes to the development of PND, and inhibiting M1 polarization of microglia may be a potential therapeutic strategy for neuroinflammatory diseases^[7,8]^.

Propofol, a widely used anesthetic noted for its rapid onset and minimal accumulation, is employed extensively in both anesthesia induction and maintenance, as well as in critical care. Clinical and animal studies have raised concerns about its potential to impair cognitive functions^[9]^, but the precise mechanisms remain unclear. Yang *et al*^[10]^ suggested that anesthetics, including propofol, may induce cognitive dysfunction by promoting microglial activation and subsequent neuroinflammation. In addition, Pearn *et al*^[11]^ found that propofol disrupts brain homeostasis by causing abnormalities in neuroglial cells such as astrocytes and microglia, contributing to neuroinflammation. Propofol also inhibits the microglial activation marker CD11b, thereby diminishing microglial activity and impacting cognitive functions^[12]^. This evidence supports the hypothesis that propofol may contribute to cognitive dysfunction by promoting M1 polarization of microglia, which in turn causes neuroinflammation.

NGF plays a crucial role in regulating microglial functions and promoting its anti-inflammatory actions. It was found to activate multiple signaling pathways that mediate the phosphorylation of the cAMP response element-binding protein (CREB) at crucial regulatory sites. Rizzi *et al*^[13]^ identified microglia as target cells for NGF, which can enhance their phagocytosis of amyloid-beta through TrkA-mediated pathways, increase its degradation, and alleviate inflammation activated by amyloid-beta. Furthermore, NGF activates multiple signaling pathways that mediate the phosphorylation of CREB at a critical regulatory site^[14]^. Enhanced phosphorylation of CREB is associated with neuronal survival and neuroprotective mechanisms and may improve neurogenesis in the hippocampus^[15]^. A decrease in phosphorylated CREB (p-CREB) expression is linked to neuroinflammatory responses^[16]^, and propofol treatment inhibits the expression of NGF and p-CREB in microglia, leading to impaired spatial recognition memory^[17]^.

As such, we hypothesize that propofol induces PND by mediating microglial M1 polarization and promoting neuroinflammation via the NGF/CREB signaling pathway. This study aims to explore the specific mechanisms underlying this process.

## Materials and methods

### Treatment of animals

This in vivo and in vitro experimental study was conducted from February 2021 to November 2024. The work has been reported in accordance with the ARRIVE guidelines (Animals in Research: Reporting In Vivo Experiments)^[18]^. Seven-week-old C57BL/6J mice (*n* = 42) were purchased from Skebes Biotechnology Co., Ltd. and acclimated for 7 days in a controlled environment with a 12-h light/dark cycle. They were housed in polystyrene cages and had free access to water. All experiments followed ethical guidelines approved by the Medical Ethics Committee (approval number: 2021ky027). The animal grouping is presented in the supplementary materials (http://links.lww.com/JS9/D768).

### Morris water maze (MWM) test

The MWM test was used to assess spatial learning and memory in mice. Mice underwent acquisition training to find a hidden platform in water (24°C–26°C) over 5 days. If they failed to find the platform within 60 sec, they were guided to it. In the probe trial, the platform was removed, and the number of platform crossings and time in the target quadrant were recorded. After administering propofol and NGF, the test was repeated 24 h later to evaluate their effects on spatial memory. The detailed procedures are provided in the supplementary materials http://links.lww.com/JS9/D768.

### Cell culture

The BV2 mouse microglial cell line was obtained from Shanghai Anwei Biotechnology Co., Ltd. Cells were cultured in DMEM/F12 medium (Gibco) supplemented with 10% FBS (Gibco), 100 U/mL penicillin, and 100 mg/mL streptomycin neomycin (Xinmei Biotechnology Co., Ltd.) in an incubator at 37°C with 5% CO_2_. When the cells reached 80%–90% confluence, they were subcultured or seeded for further experiments. Propofol (Sigma) was diluted in DMSO solution (Solabao Biotechnology Co., Ltd.) and then further diluted in the medium to achieve specific working concentrations.

### Transfection

Small interfering RNA (siRNA) targeting NGF (si-NGF) and CREB (si-CREB) were purchased from Suzhou GenePharma Co., Ltd. Following the manufacturer’s recommendations, si-NGF and si-CREB were transfected into BV2 cells using OPTI-MEM and Lipofectamine 3000, both sourced from Thermo Fisher Scientific Inc., USA. After transfection, the cells were incubated at 37°C. RNA was extracted 48 h post-transfection, and total protein was harvested after 72 h for subsequent analyses.

### Cell counting kit-8 (CCK-8) assay

Cells were seeded in a 96-well plate and cultured for 24 h, followed by the removal of supernatant and the addition of various concentrations of propofol (Sigma Company) dissolved in 0.1% DMSO (dimethyl sulfoxide; Sigma Company) for different groups, then incubated for 4 or 24 h. After incubation, the propofol-containing medium was replaced with CCK-8 solution (Cell Counting Kit-8, Dojindo Molecular Technologies, Kumamoto, Japan) and further incubated for 1 h.

### Western blot

Hippocampal tissues and BV2 cells were lysed using RIPA buffer (Solarbio Co., Ltd.) supplemented with PMSF (Solarbio) and homogenized using a grinding instrument. The lysates were then centrifuged to remove debris. Protein concentration was measured and equal amounts of protein were loaded for SDS-PAGE. After electrophoresis, proteins were transferred to PVDF membranes (Millipore Co., Ltd.). Membranes were blocked and incubated with primary antibodies (Cell Signaling Technology, Abcam, etc.) overnight at 4°C, followed by HRP-conjugated secondary antibodies and detection with ECL solution (Guangzhou Supu Biotech).

### Quantificational real-time PCR (qRT-PCR)

Total RNA was extracted using a rapid extraction kit (Shanghai Yi Shan Biotech Co., Ltd.), and cDNA was synthesized using the Prime Script RT Reagent Kit (TaKaRa Co., Ltd.). qRT-PCR was performed with SYBR Premix Ex Taq (TaKaRa) on a quantitative PCR detection system, and the relative expression levels of genes were determined using specific primers (Shanghai Shenggong Bioengineering Co., Ltd.), which are presented in supplementary materials http://links.lww.com/JS9/D768.

### Co-immunoprecipitation (Co-IP) assays

Cells were lysed and centrifuged to obtain the supernatant, which was then incubated with either IgG (Sigma) or NGF antibody (Santa Cruz Biotechnology) at 4°C overnight. Protein A/G magnetic beads (Millipore) were added and incubated, followed by washing and elution in a low-pH buffer.

### Preparation of exogenous NGF

Murine NGF protein (20 µg) was purchased from PeproTech and prepared according to the manufacturer’s instructions. The vial was first centrifuged at 10 000 rpm for 30 s. Next, 100 µL of the recommended solvent (ultrapure water) was added to dissolve the protein to a concentration of 0.2 mg/mL. The solution was mixed by pipetting until fully dissolved. Then, 900 µL of trehalose solution was added to dilute the concentration to 20 µg/mL.

### 5-Ethynyl-2ʹ-deoxyuridine (EdU) cell proliferation experiment

Cells from the BV2 microglial cell line (Shanghai Anwei Biotechnology Co., Ltd.) were treated and then incubated with a 2X EdU working solution (Beyotime Institute of Biotechnology) in serum-free medium. Afterward, the culture medium was removed, and cells were fixed with 4% paraformaldehyde. Subsequent steps included washing with phosphate-buffered saline (PBS), permeabilization with 0.5% TritonX-100, and a series of washes before the Click reaction was performed. Finally, cells were stained with Hoechst solution and examined under a fluorescence microscope for EdU incorporation.

### Flow cytometric analysis

The flow cytometric analysis was performed after treating cells, collecting the culture medium, and terminating digestion with complete medium. Cells were centrifuged, the supernatant was aspirated, and cells were resuspended in cold PBS. Following this, cells were diluted with deionized water and combined buffer was added according to the instructions. Cells were then incubated with Annexin V/FITC (Beijing Four-Square Biotech Co., Ltd.) at room temperature in the dark, after which propidium iodide (PI) solution was added, followed by PBS before proceeding to flow cytometry detection.

### Immunofluorescence

The immunofluorescence procedure involves fixing treated cells with 4% paraformaldehyde (Wuhan Selab), permeabilizing with 0.5% Triton X-100 in PBS, blocking with protein-free solution (Sever Biotech), incubating with primary antibodies (diluted in antibody dilution solution, Wuhan Boster) overnight at 4°C, followed by secondary antibodies (Suzhou EY Labs) at room temperature in the dark, and finally staining with DAPI-containing anti-fluorescence quenching agent (Solvay Biotech) before detection under a fluorescence microscope.

### Reactive oxygen species (ROS) test

ROS detection was conducted using a commercial ROS assay kit purchased from Shanghai Bioscience Biotechnology Co., Ltd. Following treatment, cells were washed twice with PBS, and culture medium was aspirated. Each well was then incubated with 1 mL of serum-free medium containing the diluted DCFH-DA working solution. This incubation was conducted in the dark for 30 min to prevent light-induced degradation. Post-incubation, cells were washed three times with serum-free medium to remove excess DCFH-DA. Subsequently, an anti-fade mounting medium containing DAPI was added, and cells were incubated in the dark for an additional 10 min before immediate fluorescence imaging.

### Malondialdehyde (MDA) testing

MDA levels, a marker of oxidative stress, were measured using an MDA assay kit (Wuhan E-bioscience Co., Ltd., Wuhan, China). Cells were treated with propofol or subjected to NGF/CREB modulation, and the MDA content was determined spectrophotometrically at 532 nm.

### Superoxide dismutase (SOD) enzyme activity assay

SOD activity, a key antioxidant enzyme, was measured using an SOD assay kit (Biyuntian Biotechnology Co., Ltd., Shanghai, China). The activity of SOD in cell lysates was determined by monitoring the inhibition of superoxide radicals, which react with a colorimetric substrate.

### Enzyme-linked immunosorbent assay (ELISA)

The ELISA procedure involves collecting cell culture supernatant after treatment, centrifuging to remove debris, and preparing samples. Reagents (from Xiamen Lunchangshuo Biotech Co., Ltd.) are equilibrated at room temperature, and then standard and sample dilutions are added to the ELISA plate. After incubation with detection antibody and washing, a substrate solution is added and incubated before stopping the reaction with a stop solution. The absorbance at 450 nm is measured to determine TNF-α levels.

### Statistical analysis

Data were analyzed using GraphPad Prism 9. Statistical significance was determined using one-way ANOVA followed by Dunnett’s multiple comparisons test. A *P* value of less than 0.05 was considered statistically significant (*P < 0.05, **P < 0.01, ***P < 0.001, ****P < 0.0001).

## Results

### Expression of NGF and CREB in BV2 microglial cells under propofol stimulation

#### The effect of propofol on the viability of BV2 microglial cells

**Figure 1.**
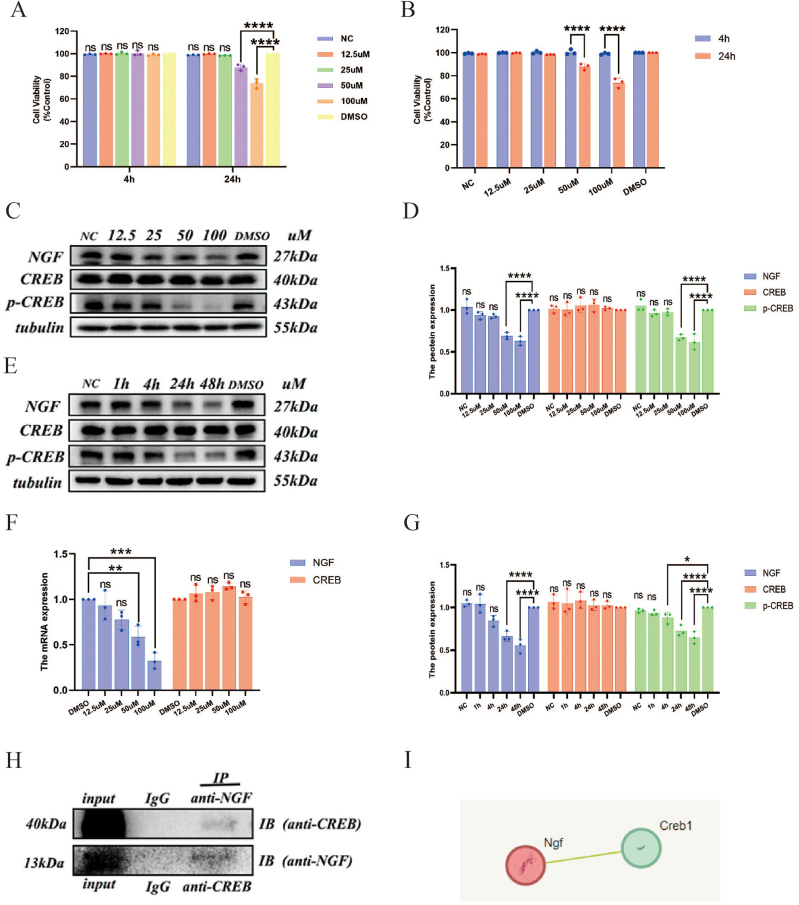
The CCK-8 assay was used to investigate the effects of propofol at various concentrations and stimulation durations on the viability of BV2 microglial cells: (A) the impact of different concentrations of propofol on cell viability at a fixed intervention duration; (B) the effect of varying intervention durations on cell viability at a fixed propofol concentration. In addition, Western blot and qRT-PCR methods were utilized to analyze the influence of different concentrations of propofol on the expression of NGF and CREB in BV2 microglial cells at various stimulation times: (C, D) the effects of different concentrations of propofol on NGF and CREB expression; (F) the impact of different concentrations of propofol on the mRNA levels of NGF and CREB; (E, G) the influence of different intervention durations on the expression of NGF and CREB. Moreover, (H) the CO-IP method was employed to verify the protein-protein interaction; (I) the relationship between NGF and CREB was explored using the string database.

We experimented with different dosages – 12.5, 25, 50, and 100 µM – administered over periods of 4 and 24 h to determine the appropriate concentration and duration of propofol intervention for this experiment. DMSO was used as a control. The results (Fig. 1A, B) indicate that at a concentration of 50 µM and after 24 h of treatment, there was a significant decrease in cell viability compared to the control group (*P* < 0.0001).

#### The impact of propofol on the expression of NGF and CREB in BV2 microglial cells

To investigate whether propofol affects the expression of NGF and CREB in microglial cells, we conducted experiments using Western blot and qRT-PCR techniques across a gradient of concentrations, with DMSO serving as the control. The findings (Fig. 1C, D, F) show that at concentrations higher than 50 μmol/L, there were significant decreases in the levels of NGF protein, NGF mRNA, and p-CREB) protein, compared to the control group (*P* < 0.01). However, the expression of total CREB protein and mRNA levels did not show significant changes (*P* > 0.05).

Using 50 μmol/L as the intervention concentration, we set up time gradients of 1, 4, 24, and 48 h, again with DMSO as the control. The results (Fig. 1E, G) indicate that NGF and p-CREB expression decreased as the duration of propofol administration increased. The differences became statistically significant when the duration exceeded 24 h for NGF (*P* < 0.0001) and 4 h for p-CREB (*P* < 0.05). Again, the expression of total CREB remained unchanged (*P* > 0.05).

#### Interaction between NGF and CREB proteins

Co-IP is a classic method used to study protein-protein interactions. The results (Fig. 1H, I) demonstrate an interaction between NGF and CREB proteins.

### Construction of vectors for transient knockdown of NGF and CREB

#### Construction of low-expression vectors for NGF and CREB and verification of transfection efficiency

**Figure 2.**
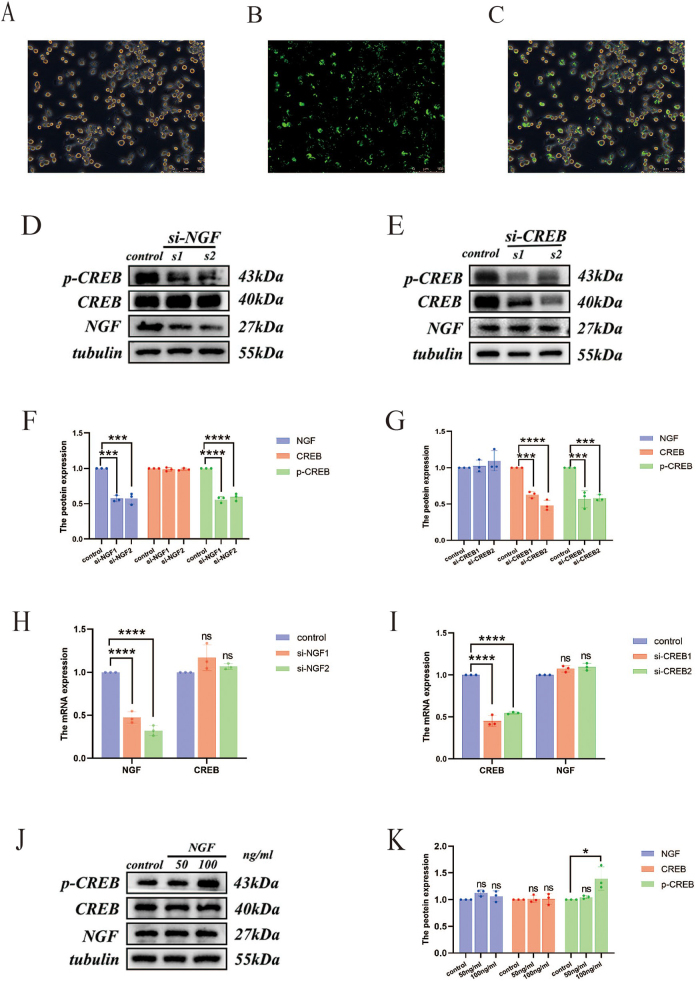
Fluorescence ratio in BV2 microglial cells after transfection: (A) bright field white light photography; (B) fluorescence ratio after transfection; (C) merge of images A and B. Western blot and qRT-PCR were used to detect changes in the levels of NGF and CREB proteins and mRNA in BV2 microglial cells after transfection (si-NGF and si-CREB represent the NGF knockdown and CREB knockdown groups, respectively). (D, F) Levels of NGF, CREB, and p-CREB proteins after NGF knockdown; (E, G) levels of NGF, CREB, and p-CREB proteins after CREB knockdown; (H) changes in NGF and CREB mRNA levels after NGF knockdown; (I) changes in NGF and CREB mRNA levels after CREB knockdown. (J, K) The effects of 50 and 100 ng/mL exogenous NGF protein on the expression of NGF and CREB in BV2 microglial cells.

Cells were transfected with fluorescent siRNA fragments to validate the transfection method and reagents’ effectiveness. Images captured under an inverted fluorescence microscope 24 h post-transfection confirmed successful transfection (Fig. 2A–C).

At 48 h post-transfection, total mRNA was extracted, and at 72 h, total protein was harvested to assess the knockdown efficiency at both transcriptional and protein levels. The control group consisted of a mock-transfected setup. The results (Fig. 2D–I) indicate that in the si-NGF1 and si-NGF2 groups, both NGF protein levels and mRNA content significantly decreased (*P* < 0.001), as did the expression of p-CREB. However, total CREB protein expression and mRNA content remained unchanged (*P* > 0.05). Similarly, for the si-CREB1 and si-CREB2 groups, both CREB protein levels and mRNA content significantly decreased post-knockdown (*P* < 0.001), with corresponding decreases in p-CREB protein levels. No significant changes were observed in NGF protein levels and mRNA content (*P* > 0.05).

### Expression of NGF and CREB in BV2 microglial cells following exogenous NGF protein intervention

#### Effect of exogenous NGF protein on p-CREB expression in BV2 microglial cells

We administered exogenous NGF protein at concentrations of 50 and 100 ng/mL and analyzed the total protein extracted 24 h post-intervention. The control group included a solvent control used to dissolve the NGF protein. The results (Fig. 2J, K) show that while exogenous NGF protein did not affect the expression levels of NGF or CREB, there was a significant increase in p-CREB expression at a concentration of 100 ng/mL (*P* < 0.05).

### NGF/CREB mediated regulation of M1 polarization in BV2 microglial cells

#### Impact of propofol and NGF/CREB knockdown on clonogenic and proliferative capabilities and induction of apoptosis in BV2 microglial cells

**Figure 3.**
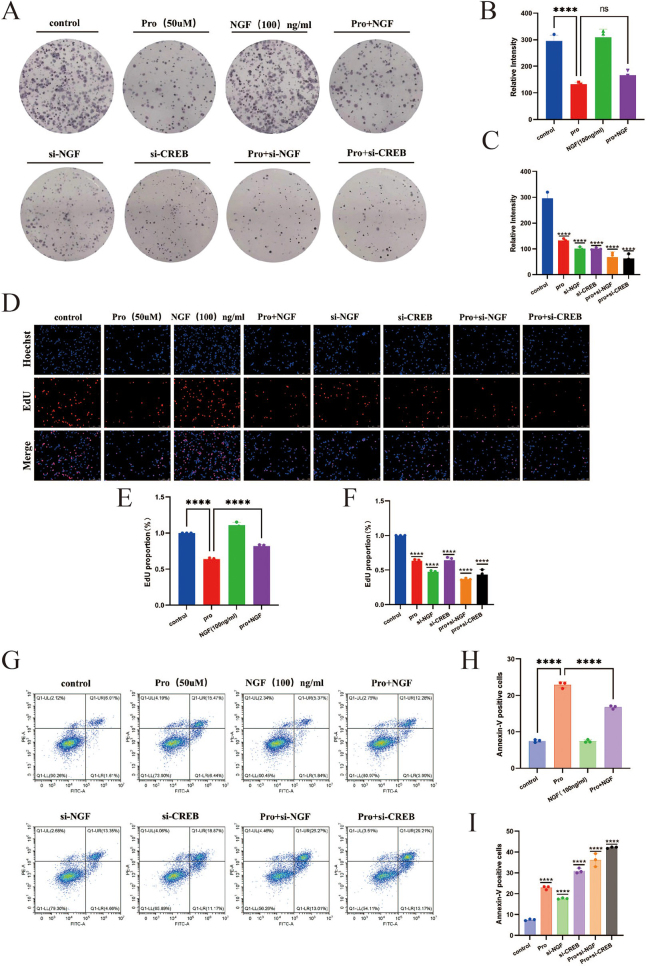
The plate cloning formation assay was used to evaluate the impact of propofol intervention, NGF/CREB knockdown, and exogenous NGF protein intervention on the clonogenic capacity of BV2 microglial cells (pro represents the propofol group): (A) the proportion of cell colonies formed after various interventions; (B, C) comparison of the number of cell colonies formed after various interventions. The EdU incorporation assay validated the effects of propofol intervention, NGF/CREB knockdown, and exogenous NGF protein intervention on the proliferation ability of BV2 microglial cells: (D) the proportion of EdU-positive cells after different interventions; (E, F) comparison of EdU content in cells after different interventions. Flow cytometry was used to verify the effects of propofol intervention, NGF/CREB knockdown, and exogenous NGF protein intervention on apoptosis in BV2 microglial cells: (G) the proportion of apoptotic cells after different interventions; (H, I) comparison of apoptotic cell counts after different interventions.

**Figure 4.**
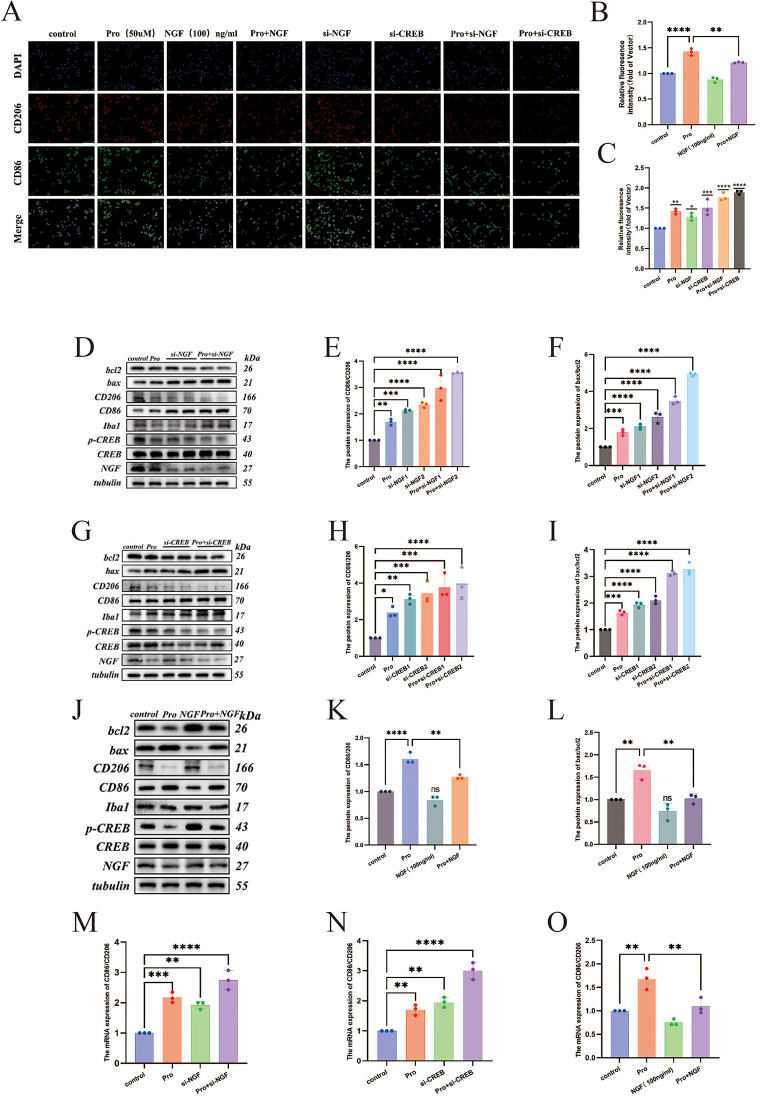
Immunofluorescence was used to assess the effects of propofol intervention, NGF/CREB knockdown, and NGF intervention on the polarization of BV2 microglial cells: (A) comparison of fluorescence intensity among groups after different interventions; (B, C) comparison of the CD86/CD206 fluorescence ratio among groups after different interventions. Western blot analysis confirmed the effects of propofol intervention and NGF knockdown on apoptosis and polarization in microglial cells: (D) comparison of various protein expression levels among groups after different interventions; (E) comparison of the CD86/CD206 protein expression ratio among groups after different interventions; (F) comparison of the Bax/Bcl2 protein expression ratio among groups after different interventions. Western blot also verified the effects of propofol intervention and CREB knockdown on apoptosis and polarization in microglial cells: (G) comparison of various protein expression levels among groups after different interventions; (H) comparison of the CD86/CD206 protein expression ratio among groups after different interventions; (I) comparison of the Bax/Bcl2 protein expression ratio among groups after different interventions. Western blot analysis was conducted to assess the effects of propofol and NGF interventions on apoptosis and polarization in microglial cells. This included comparisons of various protein expression levels across different groups after interventions (J); comparisons of the ratios of CD86/CD206 protein expression, indicative of microglial polarization states (K); and comparisons of the Bax/Bcl2 protein expression ratios, reflecting apoptotic activity (L). In addition, qRT-PCR was utilized to validate the impact of interventions with propofol, knockdown of NGF/CREB, and NGF administration on the polarization of BV2 microglial cells. This involved comparing the mRNA ratios of CD86/CD206 across different groups following various interventions (M–O), further elucidating the molecular dynamics of microglial response to these treatments.

Experiments such as plate cloning, EdU incorporation, flow cytometry, and Western blot were conducted to assess the effects of propofol intervention, knockdown of NGF/CREB, and combined propofol and NGF/CREB knockdown on the clonogenic and proliferative abilities, and apoptosis in BV2 microglial cells. The results (Fig. 3A, C, D, F, G, I, and Fig. 4D, F, G, I) indicate significant reductions in clonogenic and proliferative capabilities across the propofol, NGF/CREB knockdown, and combined treatment groups compared to the control. The ratio of proapoptotic protein Bax to anti-apoptotic protein Bcl-2 (Bax/Bcl2) increased, and the proportion of apoptotic cells rose significantly (*P* < 0.0001).

#### Induction of M1 polarization in BV2 microglial cells by propofol and low-expression NGF/CREB vectors

Further experiments including Western blot, qRT-PCR, and immunofluorescence were performed to examine the phenotype polarization of microglial cells following interventions with propofol, knockdown of NGF/CREB, and combined propofol and NGF/CREB knockdown. The results (Fig. 4A, C, D, E, G, H, M, N) showed that the ratio of M1 marker CD86 to M2 marker CD206 at protein, transcriptional levels, and fluorescence intensity significantly increased in the propofol, NGF/CREB knockdown, and combined treatment groups compared to the control (*P* < 0.01).

#### Mitigation of propofol-induced effects on microglial cell proliferation, apoptosis, and M1 polarization by NGF intervention

Experiments including plate cloning, EdU incorporation, flow cytometry, Western blot, qRT-PCR, and immunofluorescence were utilized to evaluate the effects of propofol intervention, exogenous NGF protein intervention, and combined propofol with NGF intervention on clonogenic capability, cell proliferation, apoptosis, and phenotype polarization of microglial cells. The results (Fig. 3A, B, D, E, G, H, and Fig. 4A, B, J, K, O) demonstrate that compared to the propofol group alone, the combined propofol with NGF group showed no significant difference in clonogenic capability (*P* > 0.05), increased cell proliferation (*P* < 0.0001), reduced apoptosis (*P* < 0.0001), and a decrease in the CD86/CD206 ratio at both protein and transcriptional levels, with a corresponding decrease in fluorescence intensity (*P* < 0.01).

### NGF/CREB regulation of microglial M1 polarization and induction of neuroinflammatory responses

#### Propofol intervention and NGF/CREB knockdown lead to oxidative stress in BV2 microglial cells

**Figure 5.**
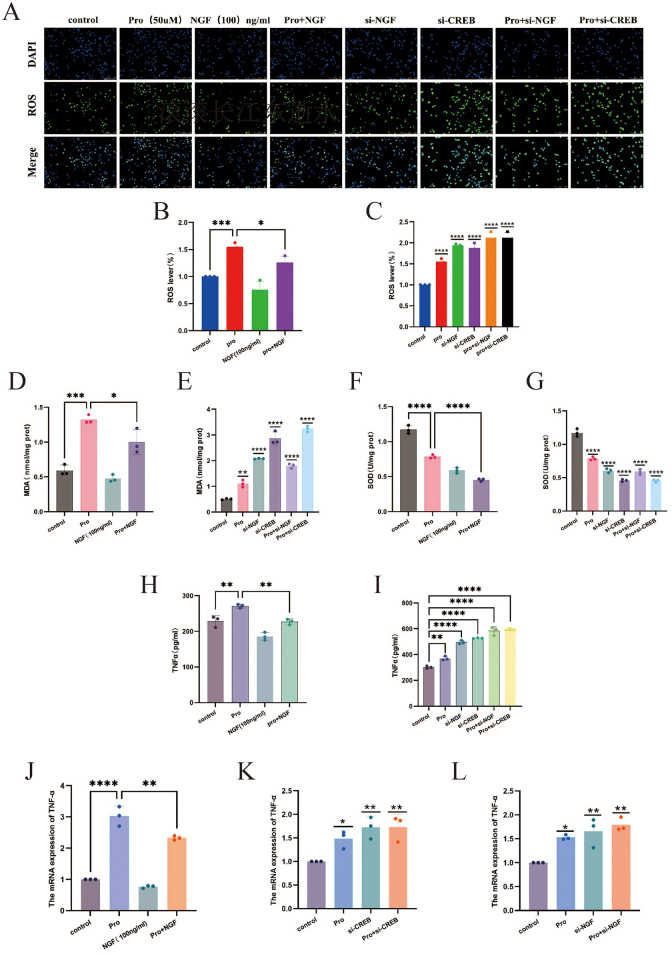
ROS detection confirmed the effects of propofol intervention, NGF/CREB knockdown, and exogenous NGF protein intervention on the ROS content in BV2 microglial cells. (A) Proportion of ROS in cells after various interventions; (B, C) comparison of ROS content among groups after different interventions. MDA and SOD assays validated the effects of propofol intervention, NGF/CREB knockdown, and exogenous NGF protein intervention on the antioxidant status and cell damage in BV2 microglial cells: (D, E) comparison of MDA content in cells among groups after different interventions; (F, G) comparison of SOD content in ROS cells among groups after different interventions. ELISA assays confirmed the effects of propofol intervention, NGF/CREB knockdown, and NGF intervention on the expression levels of TNF-α in BV2 microglial cells: (H, I) comparison of TNF-α content among groups after different interventions. qRT-PCR was used to verify the impact of propofol intervention, NGF/CREB knockdown, and NGF intervention on the transcription levels of TNF-α in BV2 microglial cells: (J–L) comparison of TNF-α mRNA content among groups after various interventions.

To evaluate the effects of propofol intervention, NGF/CREB knockdown, and combined propofol with NGF/CREB knockdown on oxidative stress in BV2 microglial cells, assessments were made using ROS, MDA, and SOD assays. The results (Fig. 5A, C, E, G) indicated that compared to the control group, the propofol, NGF/CREB knockdown, and combined intervention groups showed significant increases in ROS and MDA levels and a decrease in SOD levels, demonstrating significant oxidative stress (*P* < 0.01).

#### Propofol intervention and NGF/CREB knockdown induce elevated inflammatory cytokine levels in BV2 microglial cells

The impact of propofol intervention, NGF/CREB knockdown, and combined interventions on the expression of inflammatory cytokines in BV2 microglial cells was assessed using ELISA and qRT-PCR. The results (Fig. 5I, K, L) revealed that, compared to the control group, the propofol, NGF/CREB knockdown, and combined intervention groups exhibited significant increases in both TNF-α protein expression and mRNA levels, indicating elevated inflammatory responses (*P* < 0.01).

#### NGF intervention mitigates propofol-induced oxidative stress and inflammatory responses in microglial cells

The effects of propofol intervention, exogenous NGF protein intervention, and combined propofol with NGF intervention on oxidative stress were further evaluated using ROS, MDA, and SOD assays. The results (Fig. 5A, B, D, F) showed that compared to the propofol group alone, the combined propofol with NGF group exhibited significantly reduced levels of ROS and MDA, and increased SOD levels (*P* < 0.05).

In addition, ELISA and qRT-PCR were used to assess the expression of inflammatory cytokines following these interventions. The findings (Fig. 5H, J) indicated that, compared to the propofol group, the combined propofol with NGF group showed significant reductions in TNF-α protein levels and mRNA content (*P* < 0.05), suggesting that NGF intervention can effectively mitigate the oxidative stress and inflammatory responses induced by propofol in BV2 microglial cells.

### Propofol induces cognitive impairment in mice by promoting microglial M1 polarization through the NGF/CREB signaling pathway

#### One single high-dose injection of propofol and repeated propofol injections impair spatial learning and memory in mice

**Figure 6.**
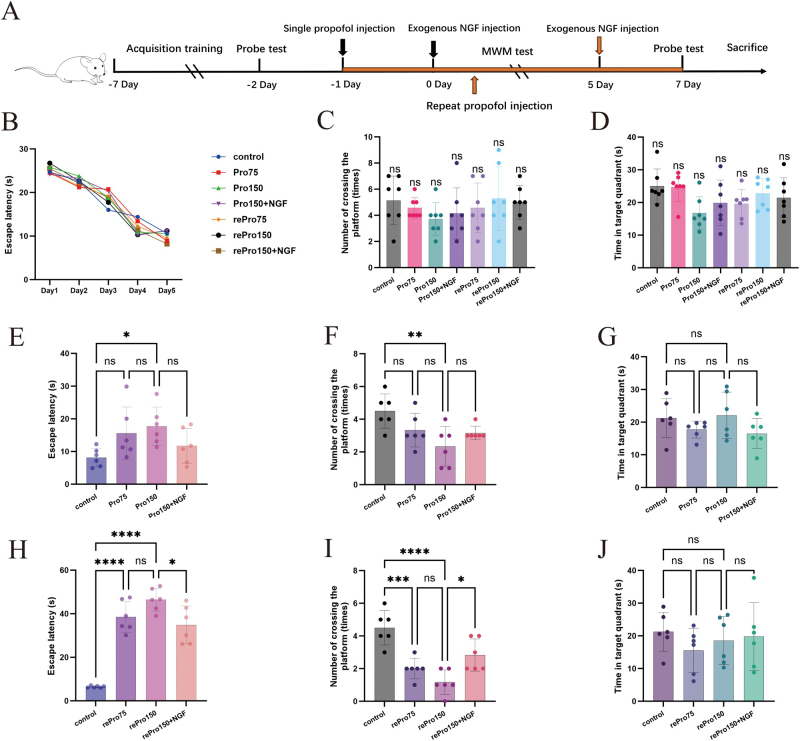
(A) Schematic diagram of the animal experiments. Spatial learning and memory abilities of mice in the MWM test before drug administration: (B) average escape latency to the visible platform during MWM testing from days 1 to 5 prior to drug administration; (C) number of platform crossings during MWM testing prior to drug administration; (D) time spent in the target quadrant during MWM testing prior to drug administration. The effects of propofol on cognitive function in mice under different treatments: (E) escape latency to the visible platform during MWM testing after a single injection of different doses of propofol or propofol combined with NGF intervention; (F) number of platform crossings during MWM testing after a single injection of different doses of propofol or propofol combined with NGF intervention; (G) time spent in the target quadrant during MWM testing after a single injection of different doses of propofol or propofol combined with NGF intervention; (H) escape latency to the visible platform during MWM testing after repeated injections of different doses of propofol or propofol combined with NGF intervention; (I) number of platform crossings during MWM testing after repeated injections of different doses of propofol or propofol combined with NGF intervention; (J) time spent in the target quadrant during MWM testing after repeated injections of different doses of propofol or propofol combined with NGF intervention.

All mice underwent five consecutive acquisition training sessions, with the experimental timeline illustrated in Figure 6A. Behavioral test results (Fig. 6B) showed that escape latency progressively decreased across training sessions for all groups, with no significant differences observed between groups. Following the final training session, probe trials were conducted. The number of platform crossings (Fig. 6C, *P* > 0.05) and the time spent in the target quadrant (Fig. 6D, *P* > 0.05) did not differ significantly between groups, indicating no baseline differences in spatial learning and memory abilities before drug administration.

**Figure 7.**
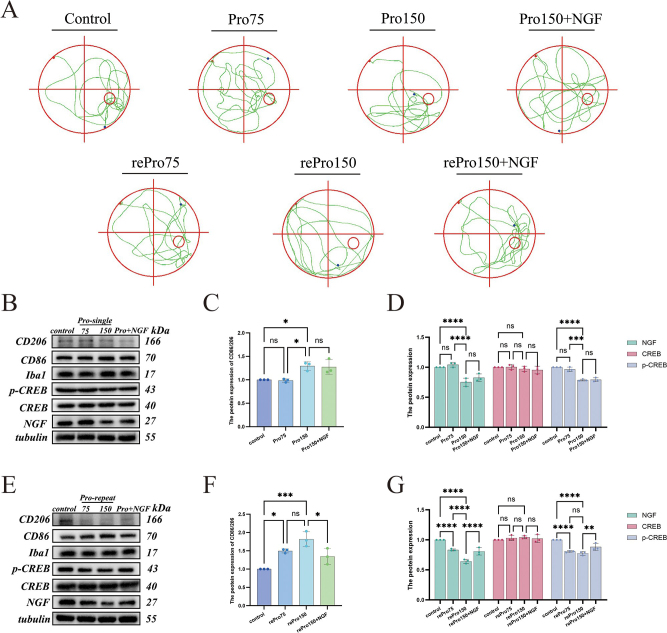
(A) Representative tracks of mice during MWM testing. (B–D) Western blot analysis showing changes in protein expression of NGF/CREB and microglial polarization markers in the hippocampus after single-dose propofol injection and co-treatment with NGF. (E–G) Western blot analysis of protein expression changes in NGF/CREB and microglial polarization markers after repeated propofol injections and co-treatment with NGF.

After completing the behavioral tests, the mice underwent the corresponding drug treatments. Behavioral tests were conducted again 24 h after drug administration for Pro75 and Pro150 groups. The results (Fig. 6E, F, G) showed no significant differences in escape latency, platform crossing frequency, or time spent in the target quadrant between the Pro75 group and the control group (*P* > 0.05). However, the Pro150 group exhibited a statistically significant increase in escape latency and a decrease in platform crossing frequency (*P* < 0.05), while the time spent in the target quadrant remained unchanged (*P* > 0.05). For the rePro75 and rePro150 groups, behavioral tests were repeated 24 h after the final drug administration. The results (Fig. 6H–J) indicated that both groups had significantly prolonged escape latency and reduced platform crossing frequency compared to the control group (*P* < 0.05), whereas the time spent in the target quadrant showed no significant difference (*P* > 0.05). Figure 7A illustrates the MWM trajectories posttreatment. These findings suggest that both a single high dose of propofol and repeated propofol injections impair spatial learning and memory in mice.

#### Exogenous NGF alleviates propofol-induced impairments in spatial learning and memory in mice

To investigate the role of NGF in mitigating the effects of propofol on spatial learning and memory in mice, NGF (100 ng/mL) was administered via hippocampal stereotactic injection in the Pro150 + NGF and rePro150 + NGF groups, followed by MWM testing. The results (Fig. 6E**–**J) showed that, compared to the Pro150 group, the Pro150 + NGF group exhibited no significant differences in escape latency, platform crossing frequency, or time spent in the target quadrant (*P* > 0.05).

However, compared to the rePro150 group, the rePro150 + NGF group demonstrated significantly shorter escape latency and increased platform crossing frequency (*P* < 0.001), while the time spent in the target quadrant remained unchanged (*P* > 0.05).

#### Propofol induces microglial M1 polarization in the hippocampus via NGF/CREB signaling, which is attenuated by exogenous NGF

The results (Fig. 7B–G) showed that, compared to the control group, there were no significant changes in the expression of NGF, CREB, p-CREB, or the CD86/CD206 ratio in the Pro75 group (*P >* 0.05). However, in the Pro150, rePro75, and rePro150 groups, NGF and p-CREB expression levels were significantly decreased, while the CD86/CD206 ratio was increased (*P* < 0.05). There was no significant difference in total CREB expression across all groups.

Compared with the Pro150 group, there were no significant differences in NGF, CREB, p-CREB expression levels, or the CD86/CD206 ratio in the Pro150 + NGF group (*P* > 0.05). However, in the rePro150 + NGF group, NGF and p-CREB expression levels were significantly increased, and the CD86/CD206 ratio was reduced (*P* < 0.05), with no significant change in total CREB expression. These results suggest that exogenous NGF can attenuate propofol-induced M1 polarization of hippocampal microglia.

## Discussion

Our studies demonstrate that propofol intervention affects microglial activity, leading to a reduction in NGF expression levels and decreased phosphorylation levels downstream of CREB. The kinase induction domain of CREB contains multiple phosphorylation sites, which can be phosphorylated by agents such as calcium ion, calmodulin kinase, protein kinase A, and protein kinase C. Phosphorylation at the serine residue at position 133 plays a critical role in the transcriptional activity of CREB. p-CREB is involved in regulating processes such as apoptosis and neuronal regeneration^[19,20]^. The interaction between NGF and CREB is also confirmed in this study. Furthermore, NGF can activate multiple signaling pathways that mediate the phosphorylation of CREB at crucial regulatory sites^[21]^. Serum levels of phosphorylated CREB are significantly associated with the extent of cognitive impairment, and with increasing age, p-CREB levels correspondingly decrease^[22]^, and a reduction in p-CREB expression is related to neuroinflammatory responses^[23]^. These findings are consistent with our experimental results. We speculate that the impact of decreased NGF expression on microglial function might be mediated through the downregulation of p-CREB.

Following this, we conducted further experiments to determine if the inhibition of p-CREB expression would promote the polarization of microglia toward a pro-inflammatory phenotype. By constructing an NGF/CREB knockdown vector, we observed that after propofol intervention and NGF/CREB knockdown, the expression of p-CREB in microglia decreased, along with reductions in clonogenic and cellular proliferation capabilities, and an increase in apoptosis levels. The ratio of M1 marker CD86 to M2 marker CD206 increased at both the transcriptional and protein levels, and immunofluorescence experiments also showed an increased fluorescence ratio of CD86/CD206. These results preliminarily indicated that propofol induces M1 polarization in microglia, which is associated with the NGF/CREB pathway. To further validate these findings, we administered exogenous NGF protein and observed that it increased p-CREB expression, alleviating propofol-induced impairments in cell proliferation, apoptosis, and M1 polarization.

In this study, we observed that after propofol intervention and knockdown of NGF/CREB, the levels of ROS in microglia increased, the expression of SOD decreased, and the expression of MDA increased, indicating oxidative stress damage. Our experimental results also showed that following propofol intervention and NGF/CREB knockdown, the transcriptional and protein levels of TNF-α in microglia were elevated. Conversely, exogenous NGF protein reduced the oxidative stress damage caused by propofol and decreased the expression levels of TNF-α. A previous study conducted by Yao *et al*^[24]^ also found that propofol could induce oxidative stress and apoptosis in SH-SY5Y cells through the miR-363-3p/CREB axis. In addition, Ebrahimi *et al*^[25]^ reported that propofol treatment induced apoptosis in hippocampal neurons and increased TNF-α activity. The increase in oxidative stress damage and the inflammatory cytokine TNF-α in our findings further support that M1 polarization of BV2 microglia after these interventions promotes the occurrence of neuroinflammation.

To further explore the relationship between propofol-induced neuroinflammation and PND, we conducted animal-level experiments. Our findings revealed that propofol impairs spatial learning and memory in mice, with the extent of damage correlating with both the dosage and frequency of administration. Compared to single injections, repeated propofol injections caused more severe long-term deficits in spatial learning and memory. Propofol was also found to downregulate NGF and p-CREB expression in the hippocampus while increasing the CD86/CD206 ratio, consistent with the results observed in cell experiments, suggesting that propofol induces M1 polarization of hippocampal microglia. Interestingly, exogenous NGF was shown to mitigate the neurocognitive impairments and M1 polarization caused by propofol, with the protective effects being more pronounced in cases of more severe damage, which provides important evidence for the development of neuroprotective agents. These results provide strong evidence that propofol contributes to the development of PND and that NGF has the potential to alleviate the neurotoxic effects of propofol.

Current studies on the effects of propofol on microglial activation have produced varying results. Some studies suggest that propofol may have both pro-inflammatory and anti-inflammatory effects, depending on factors such as dosage, exposure time, and other variables^[26]^. For instance, research by Guan *et al*^[27]^ found that propofol exerts neuroprotective effects against lipopolysaccharide (LPS)-induced neuronal damage by improving neuroinflammation and inhibiting microglial metabolic reprogramming. In addition, propofol has been shown to protect BV2 microglial cells from hypoxia-induced inflammation and apoptosis by maintaining intracellular calcium (Ca^2+^) homeostasis and activating the JAK1/STAT3 pathway^[28]^. Thus, propofol is often considered neuroprotective^[29,30]^.

However, other studies present a contrasting viewpoint. In recent years, increasing evidence suggests that propofol may possess neurotoxic effects^[31,32]^. Chidambaran *et al*^[33]^ found that propofol can lead to abnormalities in glial cells (including astrocytes and microglia), disrupting brain homeostasis and contributing to neuroinflammation. Yang *et al*^[10]^ also reported that propofol enhances the activation of astrocytes and increases levels of neuronal nitric oxide synthase, pro-inflammatory cytokines such as IL-6, and TNF-α, leading to inflammation in the hippocampus. Zhang *et al*^[34]^ proposed that propofol induces cell death in cortical cells, hippocampal neurons, and neural stem cells. Furthermore, propofol has been shown to elevate ROS and IL-1β levels, which in turn trigger neuroinflammation^[35]^. Repeated exposure to propofol has been reported to increase IL-1β, IL-6, and TNF-α levels in the hippocampus and serum^[36]^. Li *et al* also suggested that the main mechanisms underlying propofol-induced hippocampal neuronal cell death include apoptosis, excessive production of ROS, inflammation, and mitochondrial damage^[37]^. Propofol disrupts the balance between ATP supply and demand in hippocampal neurons, increasing ROS levels while reducing SOD activity^[38]^. It also inhibits the expression of CREB, which leads to enhanced TNF-α signaling. TNF-α, as an activator of death receptors, can trigger apoptotic signaling pathways^[25]^. Furthermore, propofol has been shown to activate ferroptosis, contributing to neurotoxicity. This activation further results in changes to synaptic plasticity, decreased hippocampal neuronal vitality, reduced neuronal survival, and increased apoptosis. In addition, these effects may impair the development of neural networks, potentially persisting into adulthood and causing long-term learning and cognitive deficits^[39,40]^.

As Liu *et al*^[41]^ suggested that in the early stages of LPS-induced acute neuroinflammation, the anti-inflammatory effects of propofol posttreatment are more prominent than their neurotoxic effects. However, the neurotoxic effects persist and may contribute to later neuronal damage. In the context of preexisting neuroinflammation, propofol posttreatment exhibits anti-inflammatory properties, but the neurotoxic effects induced by propofol posttreatment following neuroinflammation deserve further consideration. Therefore, while propofol may have neuroprotective effects in pathological conditions, it also exhibits neurotoxic effects on normal tissues and cells, promoting neuroinflammation and contributing to cognitive dysfunction^[11,42]^. These observations highlight the ongoing challenges in the field, and our study may provide one potential mechanism by which propofol contributes to the development of PND. In the era of personalized medicine, further investigation into the causal relationship between propofol-mediated neuroinflammation and the development of PND, along with other underlying mechanisms, remains essential for advancing our understanding.

This study has several limitations. First, the interaction between NGF and p-CREB requires more in-depth investigation. Second, we did not construct NGF/CREB overexpression vectors to further validate the role of this pathway in the development of propofol-induced neuroinflammation.

In conclusion, propofol downregulates the expression of NGF and CREB, leading to a reduction in p-CREB levels, which induces M1 polarization of microglia, promotes the progression of neuroinflammation, and further contributes to the development of PND. This study provides a theoretical foundation for further elucidating the mechanisms underlying propofol-induced cognitive dysfunction and offers new insights for the clinical prevention and treatment of cognitive impairment.

## Data Availability

The data that support the findings of this study are available from the corresponding author upon reasonable request.
